# *In Vitro* Microbiotic Fermentation Causes an Extensive Metabolite Turnover of Rye Bran Phytochemicals

**DOI:** 10.1371/journal.pone.0039322

**Published:** 2012-06-20

**Authors:** Kati Hanhineva, Anna-Marja Aura, Ilana Rogachev, Sanni Matero, Thomas Skov, Asaph Aharoni, Kaisa Poutanen, Hannu Mykkänen

**Affiliations:** 1 Institute of Public Health and Clinical Nutrition, University of Eastern Finland, Kuopio, Finland; 2 VTT Technical Research Centre of Finland, Espoo, Finland; 3 Department of Plant Sciences, Weizmann Institute of Science, Rehovot, Israel; 4 Department of Food Science, University of Copenhagen, Copenhagen, Denmark; 5 Department of Pharmaceutics and Analytical Chemistry, University of Copenhagen, Copenhagen, Denmark; University of Wisconsin, Food Research Institute, United States of America

## Abstract

The human gut hosts a microbial community which actively contributes to the host metabolism and has thus remarkable effect on our health. Intestinal microbiota is known to interact remarkably with the dietary constituents entering the colon, causing major metabolic conversions prior to absorption. To investigate the effect of microbial metabolism on the phytochemical pool of rye bran, we applied an *in vitro* simulated colonic fermentation where samples were collected with intervals and analyzed by LC-MS based non-targeted metabolite profiling. The analyses revealed extensive metabolic turnover on the phytochemical composition of the bran samples, and showed effects on all the metabolite classes detected. Furthermore, the majority of the metabolites, both the precursors and the conversion products, remained unidentified indicating that there are numerous yet unknown phytochemicals, which can potentially affect on our health. This underlines the importance of comprehensive profiling assays and subsequent detailed molecular investigations in order to clarify the effect of microbiota on phytochemicals present in our everyday diet.

## Introduction

Humans have co-evolved with their symbiotic gut bacteria (microbiota) that are metabolically active organisms influencing host metabolism and hence health [1]–[3]. It is well established that human metabolic processes are encoded not only by the genome, but also contributed by the genetic information in microbiota resulting in transgenomic metabolism with two way crosstalk: microbiota contributing to human metabolism, physiology and gene expression, which in turn modulate microbiota. The composition of microbiota is reflected by the lifestyle, diet and genetics of the host and thus varies extensively [4], [5]. Correlations between specific range of microbiota have been implicated to e.g. susceptibility to obesity [6], [7], and predisposition to diseases like cancer and bowel diseases [8], [9]. A major metabolic impact of microbiota is on the dietary constituents, which are not absorbed in the upper intestinal tract, but are subjected to metabolism by microbiota in the colon, and thereafter enter the circulation. The microbial conversion of plant-food components involves fermentation of indigestible carbohydrates to short-chain fatty acids, providing additional energy from non-digestible intake [10]. Additionally, the microbial metabolism causes major structural transformations on the dietary phytochemicals such as the polyphenols [11]–[13].

*In vitro* colonic models have allowed the detailed analysis of metabolic activities of microbiota on specific metabolites or metabolite groups of dietary products. The *in vitro* models enable to follow the temporal metabolism of the phytochemicals by microbiota (which metabolites disappear and which emerge), whereas analysis of feces only shows the remaining metabolites that have not been absorbed, and analysis of blood or urine samples shows the absorbed metabolites that have undergone host metabolic functions. The majority of the studies in the *in vitro* colonic model have focused on specific metabolites or metabolite groups [14]–[18]. The dietary phytochemicals that have been studied in most detail are the polyphenols. Typical metabolic events include cleavage of the ester or glycosidic bond, reduction of double bonds, dehydroxylation, demethylation, decarboxylation, and ring-fission, and it is concluded that relatively small number of phenolic end products, mostly benzoic-, phenylacetic-, phenylpropionic and phenylvaleric acids with different degrees of hydroxylation are formed from relatively diverse group of phenolic metabolites [11].

During the past few years, large-scale metabolomics analyses by various techniques have provided new insights to the microbial activities allowing the comprehensive and concomitant characterization of multiple metabolite profiles. However, it has so far been mainly applied to samples of mammalian origin in various set-ups [19]–[22]. In this analysis we show the effect of colonic microbiota *in vitro* on the semi-polar metabolite pool of rye bran fractions as examined by the non-targeted LC-MS metabolite profiling. The multi-step data treatment included both chemometrics multivariate tools for general data visualization, as well as univariate comparison to demonstrate the most significant metabolite markers reflecting the metabolic shift in each of the fractions. The analysis showed the extensive impact of microbiota on the rye phytochemicals, as far majority of the metabolite signals observed in the samples taken at consecutive time points of the fermentation (0, 4, 12 and 48 hours) had alterations in the intensity, and only fraction of those metabolites are such that are previously known to exist in rye.

## Results and Discussion

### General Evaluation of the Impact of the Microbial Treatment on the Rye Phytochemical Pools

In our analysis two different fractions of rye bran were subjected to the *in vitro* colon model mimicking caecal conditions. The rye bran fractions were prepared from extruded rye bran as described earlier [23]. The extractable fraction (E) contained chromatographically purified soluble phytochemicals and the unextractable fraction (UE) resilient against enzymatic hydrolysis contained the residual matrix with bound phytochemicals. Because the E fraction was earlier observed to have low pH in the process interfering the metabolite conversions, it was applied also at a lower dose (EL) allowing more physiological pH (above 5.5) and enabling the metabolite conversions [23]. All the incubated fractions were analysed by UPLC-qTOF-MS metabolite profiling and subsequent data evaluation.

#### PCA demonstrates the metabolite shift during the in vitro fermentation

**Figure 1:**
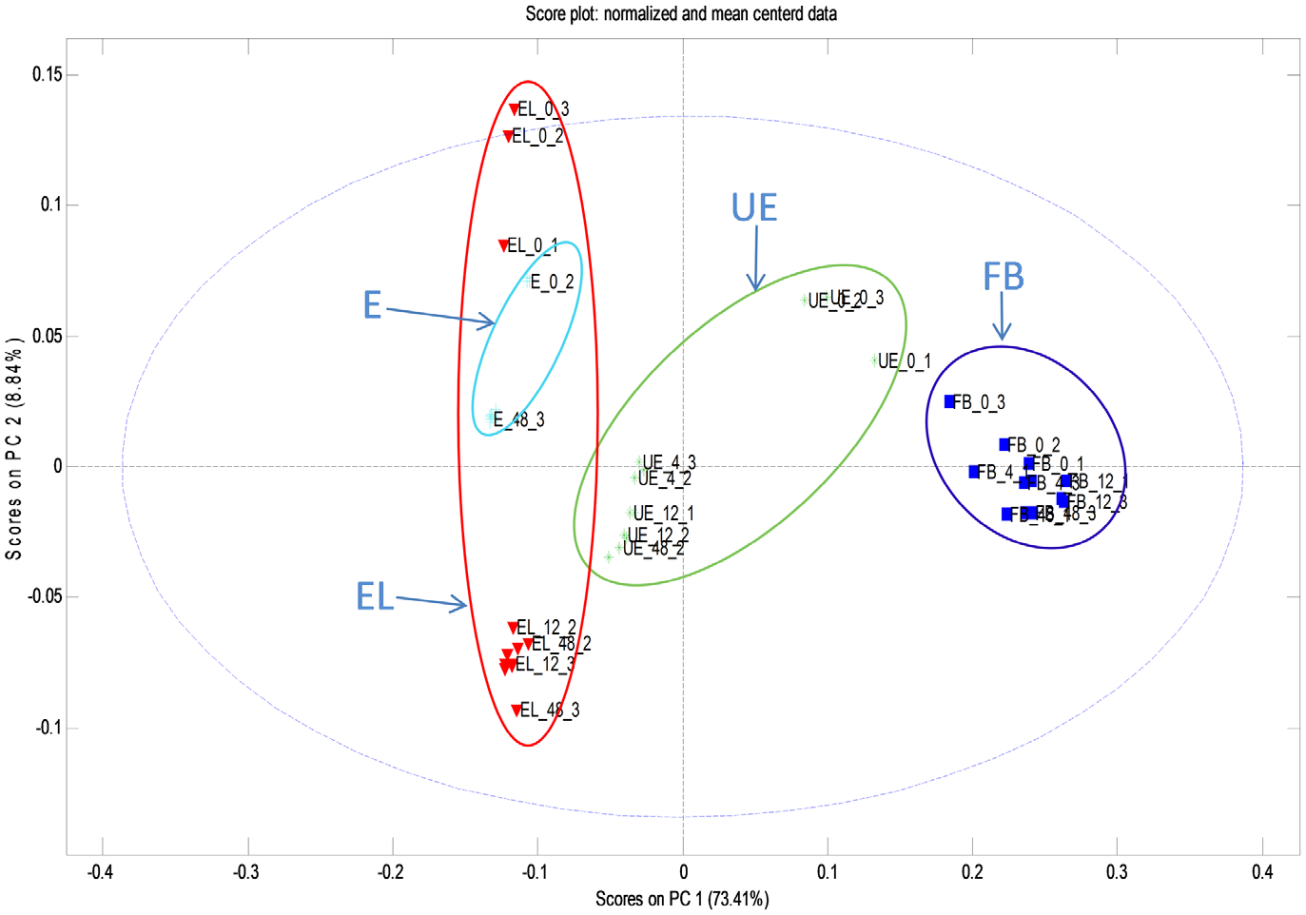
Principal component analysis, component 1 vs. component 2. The different sample types are depicted as follows: E, extractable fraction; EL, extractable fraction in low concentration; UE, unextractable fraction; FB, fecal background.

The *in vitro* simulated colonic fermentation caused extensive metabolic turnover in all the rye bran preparations studied. The effect is visible already from the total ion chromatograms of different samples and time points (Figure S1, Figure S2, Figure S3, Figure S4). Detailed examination of the metabolite signals was started by cleaning the data set from low-significance and erroneous markers resulting in set of 2147 metabolite markers. The principal component analysis (PCA) [24] brought out clear differences between sample group metabolomes as shown for the PCs 1 and 2 (Figure 1). The alterations were extensive enough to be visualized in the non-supervised manner, and there was no need to use more powerful approaches like PLS-DA [25]. First two principal components depict the differences of metabolite profiles between E(L), UE and FB samples, and show also the drift in metabolite content between the different time points. The EL samples show larger change than the E samples, indicating that the conversion process at the two different concentrations of the extractable fraction had quantitative differences (Figure 1). The larger impact of the fermentation on the EL fraction most likely resulted from the pH conditions resembling the *in vivo* conditions more closely than the E sample. Similarly as for the EL samples, also in the resilient UE fractions the pH remained favorable for the microbial conversion, and the metabolic shift is clearly visible in PCA (Figure 1).

Interestingly, metabolic changes were observed also in the faecal background samples, most likely originating from the diets of the donors of the faecal inoculum comprising of remaining dietary chemicals and their non-absorbed metabolites. The donors were prevented from eating rye, flaxseed or berries, but several plant foods, especially fruits, berries, vegetables, seeds and cereals, share common phenolic acid and lignan metabolites. Moreover, various phenolic precursors result to the same end products in the conversion by the microbiota, like phenyl propionic acid that was clearly produced in the faecal background samples [11].

### Cluster Analysis Groups Metabolite Markers with different Accumulation Patterns

**Figure 2:**
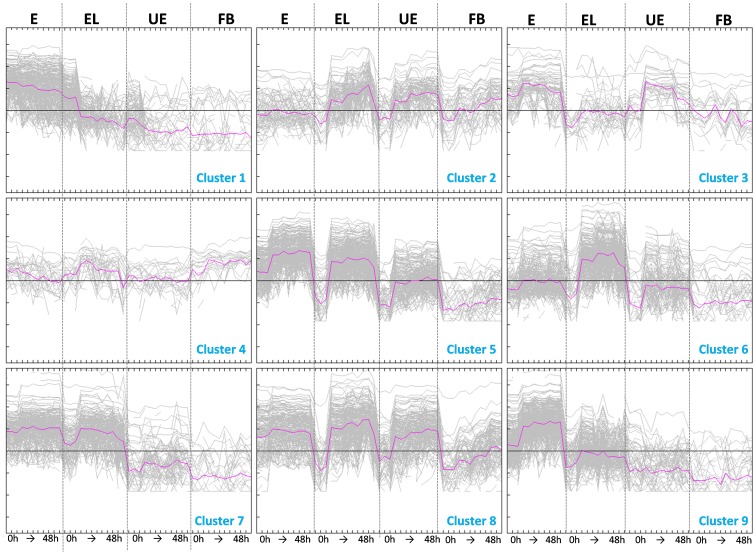
K-means cluster analysis of the cleaned data set (2147 markers). The different sample types are depicted as follows: E, extractable fraction; EL, extractable fraction in low concentration; UE, unextractable fraction; FB, fecal background, as three replicates in each sampling time (0, 4, 12, 48 h).

The cleaned marker set was next analyzed by the K-means cluster analysis in order to examine the kinetics of the different metabolite signals throughout the three fermentations. K-means clustering sorts the data by comparing each of the markers against each other, and grouping them with their nearest mean. In our data, the metabolite signals were grouped based on their presence in the different rye bran samples, their time profile (early, transient, late appearance) and strength of the metabolite signal (Figure 2). The classification of markers to a narrow set of clusters representing metabolites behaving similarly in the different sample types throughout the incubation is a valuable asset for the identification of the metabolites in a non-targeted metabolite profiling assay.

Cluster 1 contained the metabolites that are present in the samples at the onset of the fermentation, thus representing the substrates for the microbial conversions. This applies to all the three different samples (E, EL and UE) (Figure 2). All the remaining clusters represent metabolites that accumulate during the *in vitro* incubation, but differ in their proportional amounts between the different samples. It is notable that no such clear group was formed that would contain compounds that are uniquely formed in the UE samples and would not be found in the extractable fraction at all, suggesting that the differences in the phytochemical pool between the extractable and unextractable fractions are mainly quantitative, not so much qualitative.

Clusters 5 and 8 represent metabolites that are produced in all the three sample types, showing a clear increase after four hours of incubation. Many of these markers emerge also in the faecal background samples, indicating metabolites originating from the diet of the donors for the inoculums. Cluster 6 is formed from metabolites that are most clearly detected in the EL-samples, representing a trend line emerging strikingly in the 4 h samples, and slightly diminishing after 12 hours. Many of the metabolites in cluster 6 came out also in the incubation of the unextractable fraction. Similarly, markers in cluster 7 were those that accumulated initially and diminished in the incubation of the EL-fraction. These two clusters (i.e. 6 and 7) thus represented intermediate products of the bacterial incubation of the rye metabolites. Cluster 2 contained the markers that are highest at the end of the fermentation in EL- and UE-fractions, showing the final end products of the incubation. Notably, many of these metabolites are formed readily in the fecal background samples, suggesting similar metabolite profile from the diet of the donors. Cluster 9 is formed from metabolites that are accumulating early in the incubation (4 h.) in both the E and EL-samples, and then remain on relatively constant level in both samples, in higher concentration in the E than EL-fraction. Clusters 3 and 4 are the minority groups in this analysis, cluster 3 showing a peculiar metabolite group being present mostly in the E and UE fractions from 4 h. incubation onwards, but not so much in the EL fraction. Cluster 4 represents metabolites that remain in relatively constant level in all the sample types including faecal background, and are likely endogenous bacterial metabolites excreted during the incubation. One such metabolite is clearly visible also in the base peak ion chromatogram of all the sample types (*m/z* 319.19, Figure S1, Figure S2, Figure S3, Figure S4).

### Different Metabolite Families Exhibit Distinct Kinetics during the *in vitro* Incubation

From each cluster, the metabolites that showed statistically significant changes between two time points were selected for focused analysis and subjected to metabolite identification. Detailed figures on the number of metabolite markers found in each sample type are also provided in Figure S5. Only a small fraction of the addressed metabolites were such that they could be identified based on earlier reports of rye chemical composition, this being the case for both the substrate phytochemicals as well as the end products. This highlights the fact that in such non-targeted assay when metabolite repertoire is analyzed in a comprehensive manner, novel, potentially important metabolites that are not in the scope of targeted measurements can be detected. The known compounds that were detected in this study were mainly phenolic compounds of rye or their metabolites, and the unidentified compounds are presently being investigated with focused techniques for identification.

**Figure 3:**
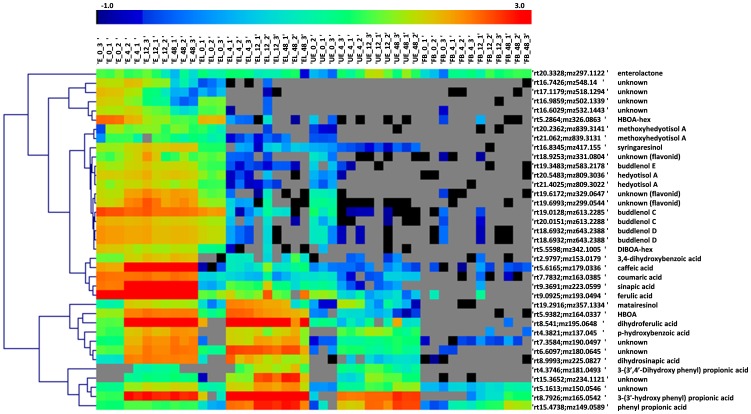
Identified and some of the most significantly changing unidentified metabolite markers sorted on heat map. The hierarchical clustering is performed with Pearson correlation. The different sample types are depicted as follows: E, extractable fraction; EL, extractable fraction in low concentration; UE, unextractable fraction; FB, fecal background followed by the sampling time (0, 4, 12, 48 h) and replicate number (1, 2, 3).

The most abundant metabolite markers are shown together with some of the most interesting unknown markers on a heat-map graph (Figure 3). Further information on the identification procedure (MS/MS fragmentation and references) is described in Table S1. We observed major alteration in the compositions of the phenolic acids (Figure 3). However, as the microbial modification of phenolic acids is discussed to large extent elsewhere [11], [15], [26], it is not addressed in detail here.

#### Lignan sub-classes were affected differentially by the microbiota

**Figure 4:**
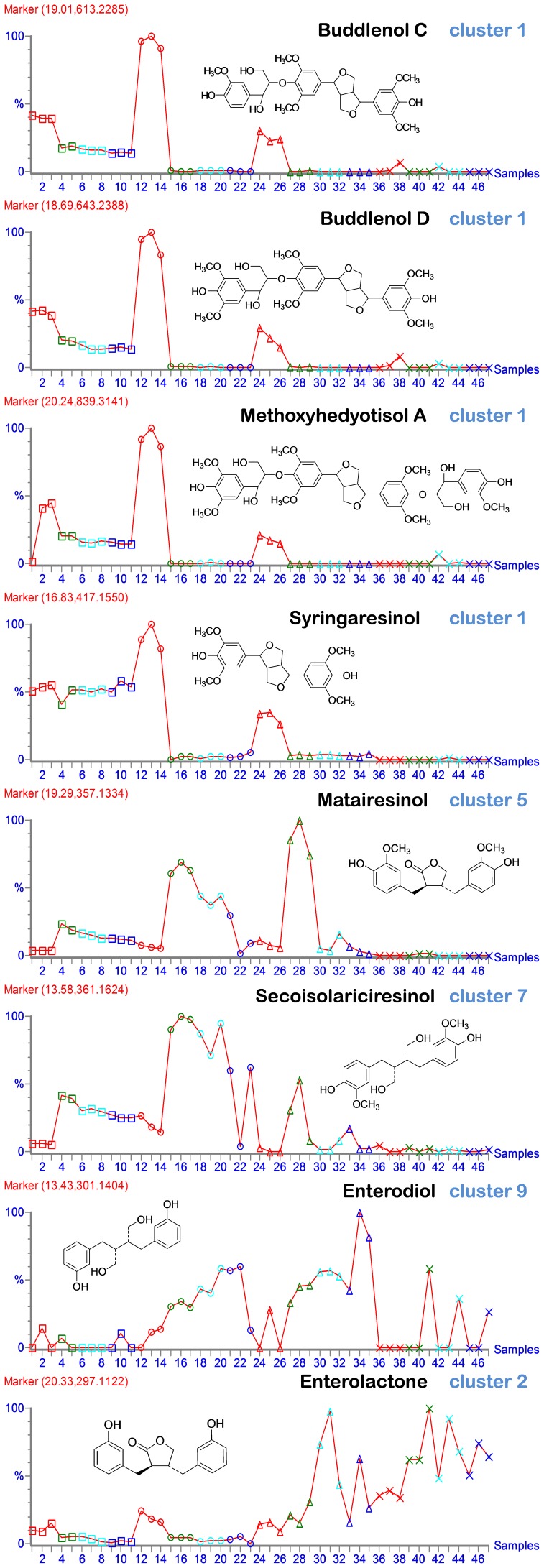
Occurrence of the identified lignan metabolites in the analysis. Three replicates from each sample type are shown; the symbols are: squares, extractable fraction (E); circles, extractable fraction in lower concentration (EL); triangles, unextractable fraction (UE); and crosses, faecal background (FB) samples. The different time points are represented with different colors: red, 0 h; green, 4 h; turquoise, 12 h; and blue, 48 h.

Our earlier analysis on rye bran lignans revealed several novel oligolignans, e.g. Buddlenol C, Buddlenol D and Methoxyhedyotisol A [27]. All of these lignans were clearly degraded in the bacterial fermentation, as their metabolite signals were decreasing after 4 h of incubation, particularly in the EL fraction in which they were reduced to trace levels (Figure 4). These lignans were also detected in the UE-fraction prior to incubation, and they likewise disappeared in the beginning of the *in vitro* treatment. Also other resinol type-lignans containing the furofuran double-ring structural moiety [28] appeared in the samples with very similar accumulation profiles, *e.g*. syringaresinol and hydroxysyringaresinol (Figure 4). All these metabolites were represented in cluster 1 in the K-means analysis. In addition, other lignan types with different pattern of accumulation *e.g.* matairesinol and secoisolariciresinol were identified (Figure 4). On contrary to the furofuran containing lignans, these were present at low levels in the beginning of incubation, but showed a transient peak after 4 h of incubation, and subsequently decreased again (in all three sample types) (Figure 4). This suggests that the furofuran-structured lignans are present as precursor lignans synthetized *in planta*, and serving as substrates for the microbial conversion and resulting in the other lignan-types. The different accumulation of the different lignan types clearly refer to separate metabolic processes most likely involving various different enzymatic reactions, potentially by different microbial species.

It has been reported that syringaresinol is the most abundant lignan in rye [29] but also other types of lignans are occurring in rye naturally without microbiota conversion, suggesting that they are also produced *in planta*, although smaller quantities than the furofuran-type resinols. The lignans that are showing intermediate accumulation in our analysis, matairesinol and secoisolariciresinol, are most likely released from the matrix or converted from other resinol-type lignans. Such metabolite conversions have been detected *in vitro* [30]. The chromatographic region containing the eluting lignans was extremely dense in metabolites as reported also earlier [27] and it included additional yet unidentified lignan-like metabolites displaying differential accumulation in the different sample types.

Microbial metabolism of plant lignans to the mammalian lignans enterodiol and enterolactone is one of the most studied microbial conversion process of dietary constituents [30]–[33]. In our analysis both of these enterolignans were detected, but in significantly smaller signals than the lignans. Enterodiol accumulated to the highest level at the EL and UE fractions at the latter stage of the incubation (24–48 hours; Figure 4), and enterolactone was detected mostly at the end of incubation in the UE samples, but also extensively in the faecal background samples. The ratio of enterolactone- and enterodiol-producing bacteria is 1∶2000 indicating that enterodiol-synthesizing bacteria are dominant while enterolactone-forming microbiota is a minor population [34]. In our case mainly the formation of enterodiol was observed in the EL and UE fractions, and enterolactone mostly only at the end of UE and FB incubation, which observations are in accordance with earlier targeted measurements of enterolignans in *in vitro* model [23], [33] as it appears that rigid structure in UE and the removal acidic components in EL favours enterolactone conversion. It is noteworthy to mention, however, that the strength of the signals for the furofuran-type lignans in this analysis was by far more intense than those for the enterodiol and enterolactone, which refers that at least in our analytical conditions the enterolignans are not produced from the plant liganas in 1∶1 ratio, and that there might be other, yet non-characterized conversion products of the lignans.

#### Benzoxazinoid aglycones are released by the microbiota

**Figure 5:**
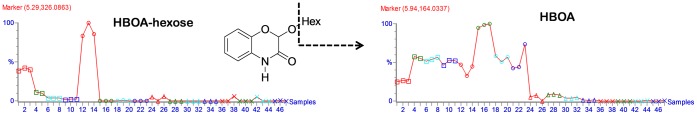
Occurrence of the benzoxazinoid HBOA-hexose and its deglucosylated form. Three replicates from each sample type are shown; the symbols are: squares, extractable fraction (E); circles, extractable fraction in lower concentration (EL); triangles, unextractable fraction (UE); and crosses, faecal background (FB) samples. The different time points are represented with different colors: red, 0 h; green, 4 h; turquoise, 12 h; and blue, 48 h.

Benzoxazinoids are relatively rare dietary phytochemicals restricted to a narrow set of plant species including rye, wheat and maize. Our earlier analyses showed that these metabolites are present in rye bran as hexose and di-hexose derivatives [35]. In the *in vitro* fermentation, the hexose containing derivatives were rapidly deconjucated, as seen for the two most abundant benzoxazinoids, DIBOA-hex and HBOA-hex (Figure 3). In contrast, the released aglycone of HBOA was visible as an intense signal after 4 h in both the E and the EL fractions. Candidate markers for the aglycone of DIBOA were detected as well; however, they could not be unequivocally identified due to co-eluting compounds with same molecular weight. In the EL sample, the fermentation continued further, as the amount of the free HBOA aglycone was reduced in the 12 and 48 h samples (Figure 5). The microbial degradation product of the benzoxazinones was not identified in this study. In the same chromatographic region, we detected several nitrogen- containing metabolites that were most abundant at the end of the fermentation (e.g. *m/z* 150; Figure 4), which could be either degradation products from the benzoxazinoids, or potentially microbially processed amino acids. Benzoxazinoids as dietary products have not yet gained much attention. The end products for this metabolite group have not been studied, and whether they can be absorbed to circulation is not known. A very recent study, however, describes that in pig feeding trial [36] benzoxazinoids are absorbed and detectable in urine and blood postprandially, which suggests that they may be bioavailable in humans as well.

#### Large amount of rye bran phytochemicals and their microbial conversion products remain to be identified

One of the largest groups represented in the cluster analysis were metabolites that are degraded during the incubation (see cluster 1; Figure 2). Based on earlier studies we were able to identify many of those, yet, several of the most significantly changing metabolites with very high intensity were not precisely annotated. These molecules included three closely eluting peaks (*m/z* 331.08, 329.06, 299.05) (Figure 3), possibly flavonoid structures, as the survey for potential molecular formulae pointed solely to flavonoids, mainly flavonols and flavanols, and their UV spectra resembled a typical flavanol absorbance. The flavonoids of rye bran in general are not studied and reported in detail, and thus the structural identity of these metabolites will be analyzed in a future study.

A particularly interesting group of unknown peaks possibly containing nitrogen and most likely represent related metabolites was observed in the analysis (*m/z* 532.14, 548.14, 502.13, 518.13) (Figure 3). These compounds eluted in the same region and their molecular formulae referred to the same class of indole containing compounds. All of these were found only in the extractable fraction (both E and EL), and were rapidly degraded in both samples in the incubation. Similarly, also various smaller nitrogen containing metabolites accumulated as end products (*m/z* 150.05, 190.05, 234.11) (Figure 3). Those resemble metabolites that have been detected in human plasma as microbial conversion process of dietary tryptophan [19]. In the case of the rye fractions, these metabolites could not be identified based on MS spectral properties, but will be addressed with targeted approach has been used for the structural resolving of phytochemicals [37].

The last few minutes in the chromatographic separation eluted hydrophobic metabolites such as lipids. In the rye bran fractions this region was relatively rich in metabolites, and also here different accumulation patterns were observed for individual peaks (*m/z* 329.23, 357.26, 229.24, 373.26), suggesting modification by the colonic microbiota. Although the metabolites in this region were not identified one by one, the characteristic increase of 2 amu in the molecular ion reflects to the reduction of carbon-carbon double bonds that is generally performed by microbiota on e.g. polyphenols. The hydrophobic faction will be taken into further analysis with non-polar extraction solvents and chromatographic conditions (lipidomics).

### The Applicability of *in vitro* Metabolomics Analysis in the Nutritional Research

Colon with its microbiota functions as a bioreactor with metabolic capacity far beyond that of the human metabolism. Large metabolome-level analyses on the effect of microbiota on the dietary phytochemicals are scarce, as most of the studies have focused on the detailed analysis of single or few compounds. However, the large-scale *in vitro* studies from whole food items provide important addition when the fate of dietary phytochemicals is examined, as they allow distinguishing between metabolites originating from microbial conversion from the ones produced by host metabolism [38]. The information of microbial metabolic conversions on dietary phytochemicals is especially valuable, when it is connected with nutritional post-prandial or intervention studies. When unknown molecules from similar non-targeted profiling arrays appear from human derived samples, the analysis of microbiota treated samples (from the same or similar dietary item or raw material) will help identification of the *in vivo* metabolites, and allow distinguishing whether they are native plant made metabolites or microbial conversion products. The simple metabolite extraction and the same analytical conditions hold true for all sample types in the non-targeted metabolomics assay, enabling effective parallel profiling. Similarly, multi-level non-targeted profiling can be applied on raw material, on processed foods, and also mammalian samples (biofluids from humans, tissues from animal feeding trials), as well as on temporal sample set providing straightforward approach for nutrikinetics studies. For example in our approach, we could show that the benzoxazinoid metabolites are processed by microbiota – firstly released from the sugar and also seemingly undergo modifications on the aglycon part as well. The fact that we had recently analysed the benzoxazinoids from the starting material (the rye bran) with the very same analytical approach enabled easy identification of those metabolites. Furthermore, these observations raise a totally new branch of research – targeted analysis of the benzoxazinoid metabolism as dietary metabolite family.

The metabolomics analyses on the effect of microbiota have mainly been done in human or animal models, with the NMR profiling experiments dominating the field [39]). Although NMR gives a relatively broad, quantitative profile of *e.g*. main blood metabolites, it fails in detecting low concentration molecules. LC-MS based analytics offer more sensitive approach to monitor also the diet derived phytochemicals, many of which are found in circulation in low levels, but yet may exert important bioactive capacity that needs to be taken into account when the effect of diet on health is addressed. The major bottleneck limitation of large-scale LC-MS profiling studies is the lack of tools for clear cut identification of the measured metabolite signals. Compound collections with MS spectral information such as human metabolome database [40] are gradually gathering up information mainly on endogenous human metabolites, easing the interpretation of human plasma and urine derived MS-data. However, the metabolites harboring our body via food, especially plant-based products, multiply the number of metabolites present endogenously by several factors. As the majority of phytochemicals present in our everyday food are still uncharacterized, the detection of unknown metabolites in any wide scale MS analysis is foreseen still for quite some while. On the other hand, the utility of non-targeted LC-MS profiling in bringing out previously non-characterized metabolic components is outweighing, as it takes into account all the chemical information in the taken sample, in contrary to targeted measurements that overlook any other compounds than the focused ones.

In our study, the number of compounds detected in the rye bran fractions was very large, and further increased by the bacterial transformations, resulting in an overwhelming myriad of chemicals, (that, theoretically, could be bioavailabe and enter circulation). Even many of the metabolites present in most abundant signals were previously non-characterized in the source material (rye bran), which underlines the importance of non-targeted profiling arrays in bringing out novel chemical information. Our analysis showed the importance of colonic conversions in modulating the diet derived chemical species, and demonstrates the applicability of MS profiling for large scale metabolite screening analyses as first step in analysing dietary phytochemical composition, which is pre-requisite for the investigation of the relationship between diet and human health status.

## Materials and Methods

### Ethics Statement

At the time of the performance of the presented colon model experiment, the Ethical Committee of VTT Technical Research Centre of Finland was not involved, because the research do not fill the criteria of medical research and donation of feces was a non-invasive method and results were not obtained on personalized level. Volunteered donors of the inoculum were informed orally and the origins of the faecal samples were shielded by number coding and key was known only by one technician and responsible scientist (Aura). All results were expressed per pooled faecal inoculum with no direct reference to a particular person. The voluntary donors of the faeces belong to a panel and have given their consent orally before 2010, received oral and written information on the studies regularly and have a possibility to refuse anytime from participation. Ethical Committee of VTT Technical Research Centre of Finland confirmed in their statement in 2010 that no permission is needed according to the applicable law, however, a written consent was recommended and applied instead of oral change of information and consent. After obtaining the statement and guidelines from Ethical Committee of VTT in 2010, all donors of the panel have signed an informed consent and the same principals as above are approved and confirmed as adequate measures as far as the ethics are concerned.

The VTT colon models have been applied in several projects, which were audited by institutional quality and safety authorities, the Safety-quality panel and biological hazard authorities of VTT. Faeces from health volunteers are not classified in the worst hazard level in terms of pathogenic bacteria and all personnel working with feaces have been vaccinated against polio, tetanus and hepatitis A and B. All possible laboratory ware is disposable. Desinfectant effective also on viruses (Virkon) is used for non-disposable ware and instructed laboratory personnel take care of the wastes.

### In Vitro Fermentation of Rye Bran

The extractable fraction was released from the rye matrix in the enzymatic hydrolysis of the extruded rye bran, and the soluble clear supernatant was bound to the column, eluted, dried and applied in the model in two concentrations. The unextractable residue is the core rye matrix resistant to the hydrolysis, but degradable by the microbiota releasing the phytochemicals for further conversions.

Bran samples of rye (*Secale cereale*) from Finnish origin were used for the study. The extractable fraction was released from the rye matrix in the enzymatic hydrolysis of the extruded rye bran and separated from the core unextractable rye matrix resistant to the hydrolysis. The soluble fraction was further purified chromatographically and dried to a phenolic-rich powder [41]. Both fractions were introduced to the colon model as described previously [42] in two experiments with following modifications: Fecal material was collected from four donors (enhances the diversity of the microbiota and evens up the differences in the conversion activities of the microbial populations), who had not received antibiotics for at least 5 months, and had not eaten flaxseed, rye or berries for at least 3–4 days before donation. Freshly passed feces was immediately transferred to a strictly anaerobic chamber for further processing of the fecal suspension 16.7% (w/v) by homogenizing the feces in 0.22 M carbonate −0.02 M phosphate buffer [43].

The water-extractable, soluble fraction of the phytochemicals was used in two different concentrations, 1 g per 10 ml of inoculum, termed as extractable fraction (E) and 0.25 g per 10 ml of the inoculum, termed as extractable sample in lower concentration (EL), at initial pH values of 7.14±0.02 and 7.63±0.03, respectively. The lower dose was tested, because our earlier analyses showed that some of the phytochemical conversions can be suppressed when high dose of the polyphenolic mixture was applied [23]. The residual fraction was applied in one concentration (1 g/10 ml) and was termed as unextractable fraction (UE). Additionally, the metabolomics analysis was performed on the faecal background samples (FB) without addition of rye bran extracts, to be able to identify the metabolites originating from the diet of the donors of the faecal inoculum.

### Metabolite Extraction

The bacterial cells were removed from the samples as follows: 100 mg of freeze-dried *in vitro* fermentation samples were suspended in saline (0.9% NaCl) in a ratio 9 µl of saline per mg sample in triplicates. The suspension was centrifuged (2500 rpm 10 min) and a 1.5 ml aliquot of the supernatant was moved to eppendorf tube and centrifuged once more (14 000 rpm 10 min). 500 µl of the supernatant was filtered (PTFE 0.4 µm), extracted with equal amount of ethyl acetate, evaporated to dryness under nitrogen flow, and stored in −20°C. Upon UPLC-qTOF-MS analysis the samples were redissolved in 200 µl of 75% MeOH +0.1% Formic acid and filtered (PTFE 0.2 µm), and 4 µl of sample was injected.

### UPLC-qTOF-MS Analysis

Metabolite analysis was carried out using a UPLC-qTOF-MS system (Waters Acquity UPLC and Synapt HDMS in the standard qTOF mode). The column was a 100×2.1 mm i.d., 1.7 µm UPLC BEH C18 column (Waters Acquity). The mobile phase consisted of 0.1% formic acid in acetonitrile: water (5∶95, v/v) (phase A), and 0.1% formic acid in acetonitrile (phase B) in a linear gradient: 100–72% A over 22 min, 72–60% A over 0.5 min, 60–0% A over 0.5 min, held at 100% B for a further 1.5 min, then returned to the initial conditions (100% A) in 0.5 min, and conditioning at 100% A. The flow rate was 0.3 ml/min; column temperature was kept at 35°C. Ionization was performed with electrospray ionization (ESI) in the positive and negative modes. The following settings were applied during the LC–MS runs: capillary voltage at 3.0 kV; cone voltage at 30 eV; collision energy at 3 eV and at 20 eV; argon was used as collision gas. For the LC–MS/MS analysis 20 and 35 eV collision was used. The *m/z* range was 50–1500 Da. The MS was calibrated using sodium formate, and leucine enkephalin was used as the lock mass. The MassLynx software version 4.1 (Waters) was used to control all instruments and calculate the accurate masses.

A standard mixture was used to monitor the quality of the chromatogram and reproducibility of the retention time throughout the runs and to aid in metabolite identification, and contained 40 µg/ml of each of the following compounds: L-tryptophan, L-phenylalanine, p-coumaric acid, caffeic acid, sinapic acid, benzoic acid, quercetin dehydrate, kaempferol, rutin, and trans-resveratrol (all purchased from Sigma); naringenin, chlorogenic acid hemihydrate, trans-cinnamic acid and isorhamnetin (Fluka), ferulic acid (Aldrich) and tomatine (Apin chemicals). Additional standard mixture of small phenolic metabolites and lignans was included in the LC-MS analysis which contained secoisolariciresinol, lariciresinol, pinoresinol, and matairesinol (Arbonova, Turku, Finland); *p*-hydroxybenzoic acid, 3,4-dihydroxybenzoic acid, vanillic acid, 3-phenylpropionic acid, and (*S*)-3-hydroxy-3-phenylpropionic acid (Sigma).

### Data Analysis

The chromatograms obtained from UPLC-qTOF-MS analysis were processed by the MarkerLynx 4.1 software (Waters) for mass signal extraction and alignment. To remove false zero values from the data matrix following rules based on signals of replicate measurement were applied: [1] if metabolite values are zero in more than 50% of the replicates and the non-zero values are smaller than the threshold (default intensity  = 5), then zero is considered a true zero and replicate values are replaced with zeros; [2] if metabolite values are zero in more than 50% of the replicates and the non-zero values are higher than the threshold (default intensity  = 5), then zero is considered false and is replaced with a “missing value”; [3] if metabolite signals of zero are present in less than 50% of the replicates, then zero is considered false and replaced with the average value of non-zero reference values. Next, data matrix was reduced by removing the very low-intensity metabolite values, which are not amenable for identification in non-targeted profiling assay. This was done by omitting markers that had maximum intensity values lower than 10, resulting in data set of 2147 metabolite signals for further analysis. Data handling was carried out by MATLAB and PLS_Toolbox (version 5, Eigenvector Inc. Seattle, WA) for unscaled data.

Principal component analysis (PCA) was applied for visualization of the pattern of different treatments and to recognize the most important markers signaling the differences between time points [24]. PCA decomposes the original data into a set of new (latent) variables (utilizing the common variances in the data) that are linear combinations of the original variables, also called Principal Components – PCs –. These new variables, PCs, exhibit two different characteristics: scores, that depicts the position of objects (treatments) in the new variable space; and loadings, that are weighted positions of original variables (markers) in the new variable space. Thus by exploring scores object patterns in the data and trends can be studied (how objects are; similar or different) whereas exploring loadings the variables accounting for those trends can be spotted (why objects are similar or different).

K-means cluster analysis and hierarchical clustering were performed by the open-source software Multi experiment Viewer (MeV, http://www.tm4.org/ [44]). These were applied to visualize the common trends in the temporal profile of the different metabolite markers (K-means clustering) as well as visualizing the abundance of the markers when compared against the other markers present in the same cluster. The number of clusters was set to nine.

The identification of metabolites was performed as described previously [45]. In short, if commercial standards were included in the analysis, the retention time and MS/MS fragmentation were used as basis for identification. For other metabolites, the accurate mass and molecular formula predictions were screened of putative molecules from the Dictionary of Natural Products (Chapman & Hall/CRC), SciFinder Scholar database (SciFinder ScholarTM 2007), and ChemSpider chemical database (http://www.chemspider.com/). The MS/MS fragmentation of the metabolites was compared with candidate molecules found in databases, and verified with earlier literature on same or similar compounds.

## Supporting Information

Figure S1 **The base peak ion chromatogram of extractable fraction in ESI(-) MS after 0, 4, 12, and 48 hours incubation.**
(TIF)

Figure S2 **The base peak ion chromatogram of extractable fraction with low concentration in ESI(-) MS after 0, 4, 12, and 48 hours incubation.**
(TIF)

Figure S3 **The base peak ion chromatogram of unextractable fraction in ESI(-) MS after 0, 4, 12, and 48 hours incubation.**
(TIF)

Figure S4 **The base peak ion chromatogram of faecal background in ESI(-) MS after 0, 4, 12, and 48 hours incubation.**
(TIF)

Figure S5 **Number of metabolite markers, and the proportion of significantly changing markers found in each sample type.** (**A**) The number of metabolite markers in each of the samples types after the data cleaning process, and number of significantly decreasing and increasing markers in each sample type. Marker was considered to be significantly changed if the value between two time points had fold change either <0.5 or >2, with *p*-value less than 0.05. (**B**) The distribution of the increasing (upper pie charts) and decreasing (lower pie charts) markers in the incubation after 4, 12, and 48 hours. Some of the markers were intermediate metabolites that were first increasing, but towards the end of the incubation were again decreasing, and these are shown on purple sectors in the upper pie charts.
(TIF)

Table S1 **MS/MS fragmentation of the key metabolites.** Identification was done based on comparison with standards, or with earlier reported MS/MS results.
(DOCX)
