# Supplementary figures and images for: In Vitro Microbiotic Fermentation Causes an Extensive Metabolite Turnover of Rye Bran Phytochemicals
Source: PLoS One. 2012 Jun 20;7(6):e39322. doi: 10.1371/journal.pone.0039322 (PMC3380017; doi:10.1371/journal.pone.0039322)

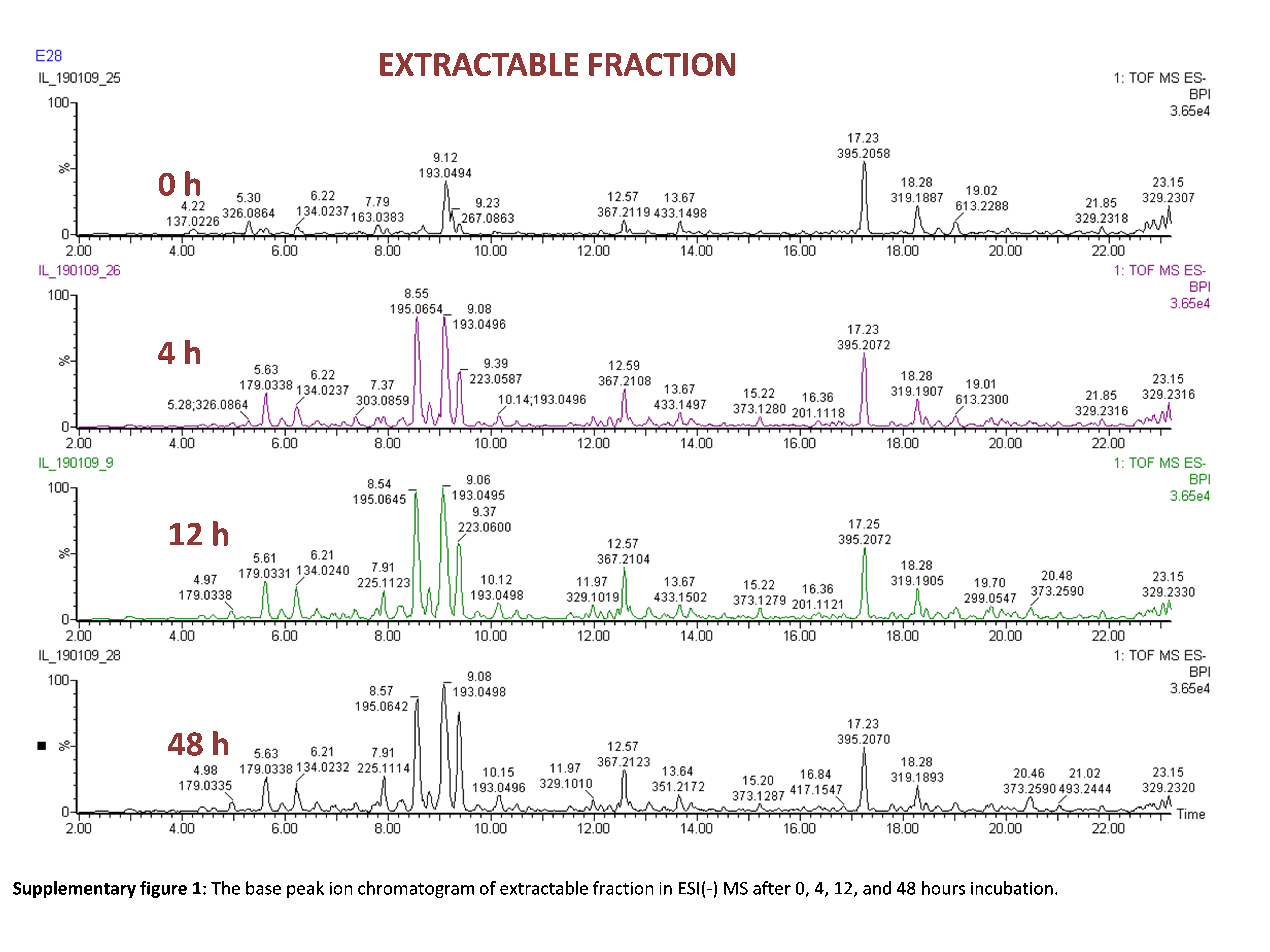

Supplement: Figure S1 — The base peak ion chromatogram of extractable fraction in ESI(-) MS after 0, 4, 12, and 48 hours incubation. (TIF) [file pone.0039322.s001.tif]

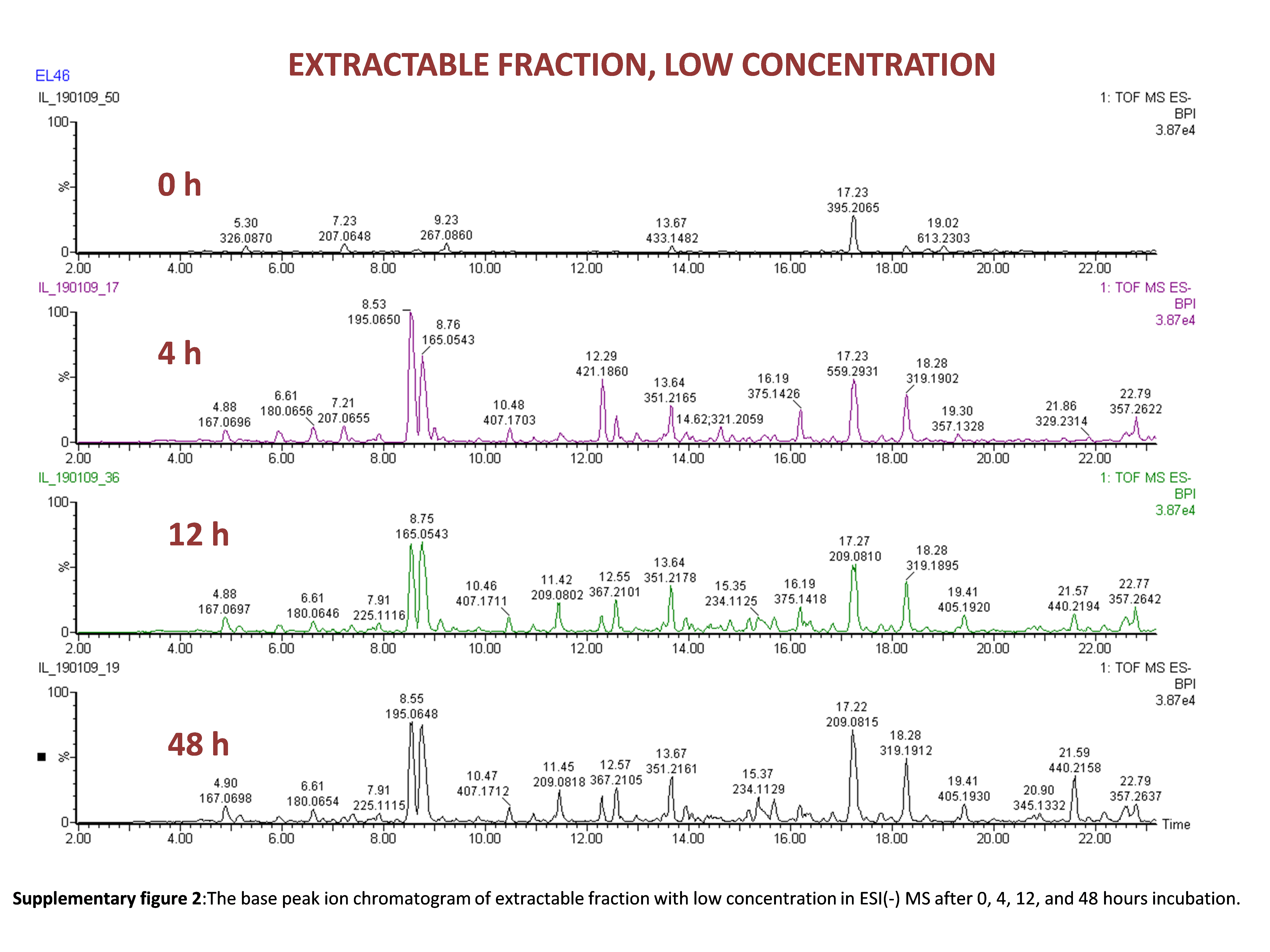

Supplement: Figure S2 — The base peak ion chromatogram of extractable fraction with low concentration in ESI(-) MS after 0, 4, 12, and 48 hours incubation. (TIF) [file pone.0039322.s002.tif]

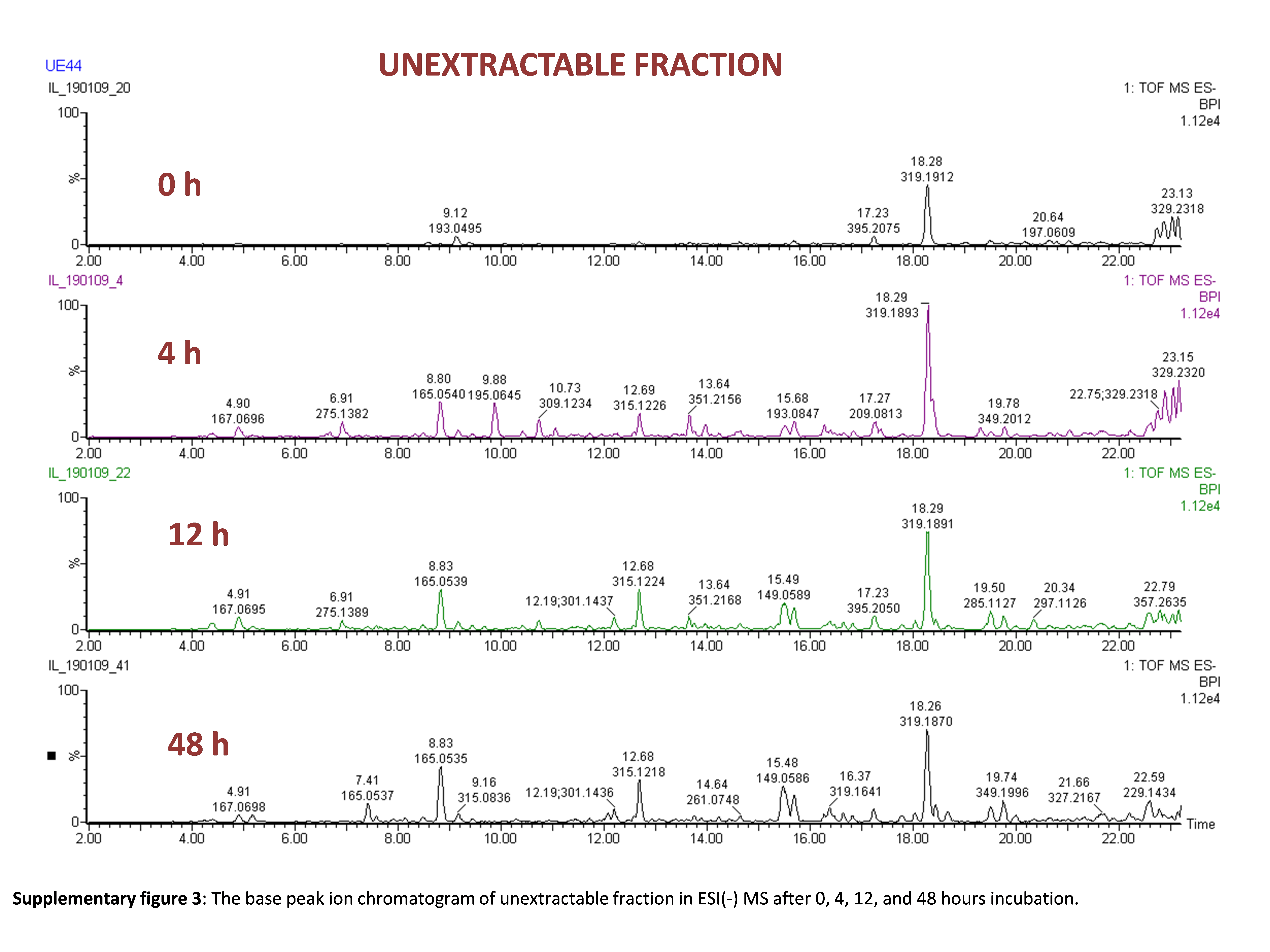

Supplement: Figure S3 — The base peak ion chromatogram of unextractable fraction in ESI(-) MS after 0, 4, 12, and 48 hours incubation. (TIF) [file pone.0039322.s003.tif]

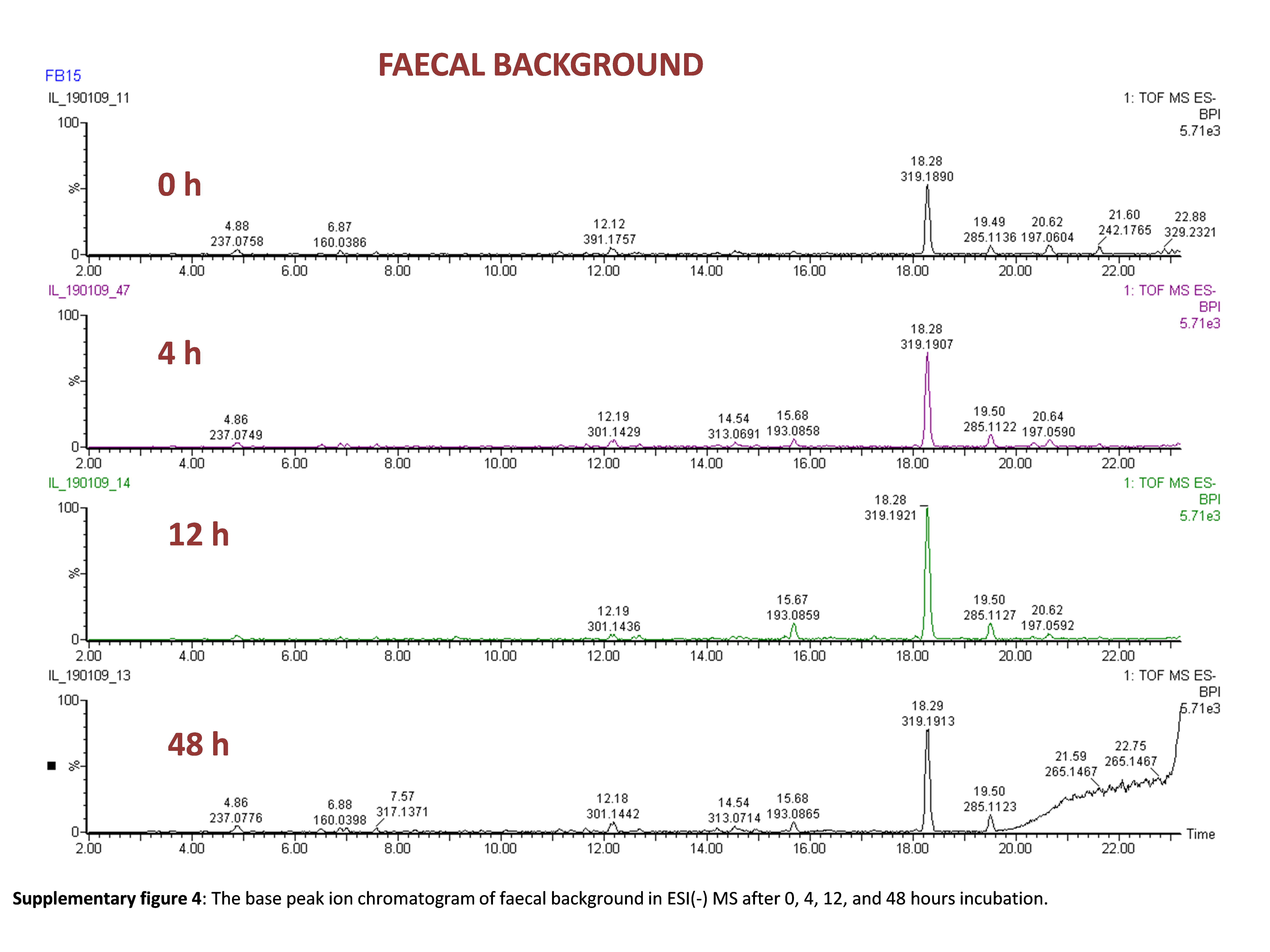

Supplement: Figure S4 — The base peak ion chromatogram of faecal background in ESI(-) MS after 0, 4, 12, and 48 hours incubation. (TIF) [file pone.0039322.s004.tif]

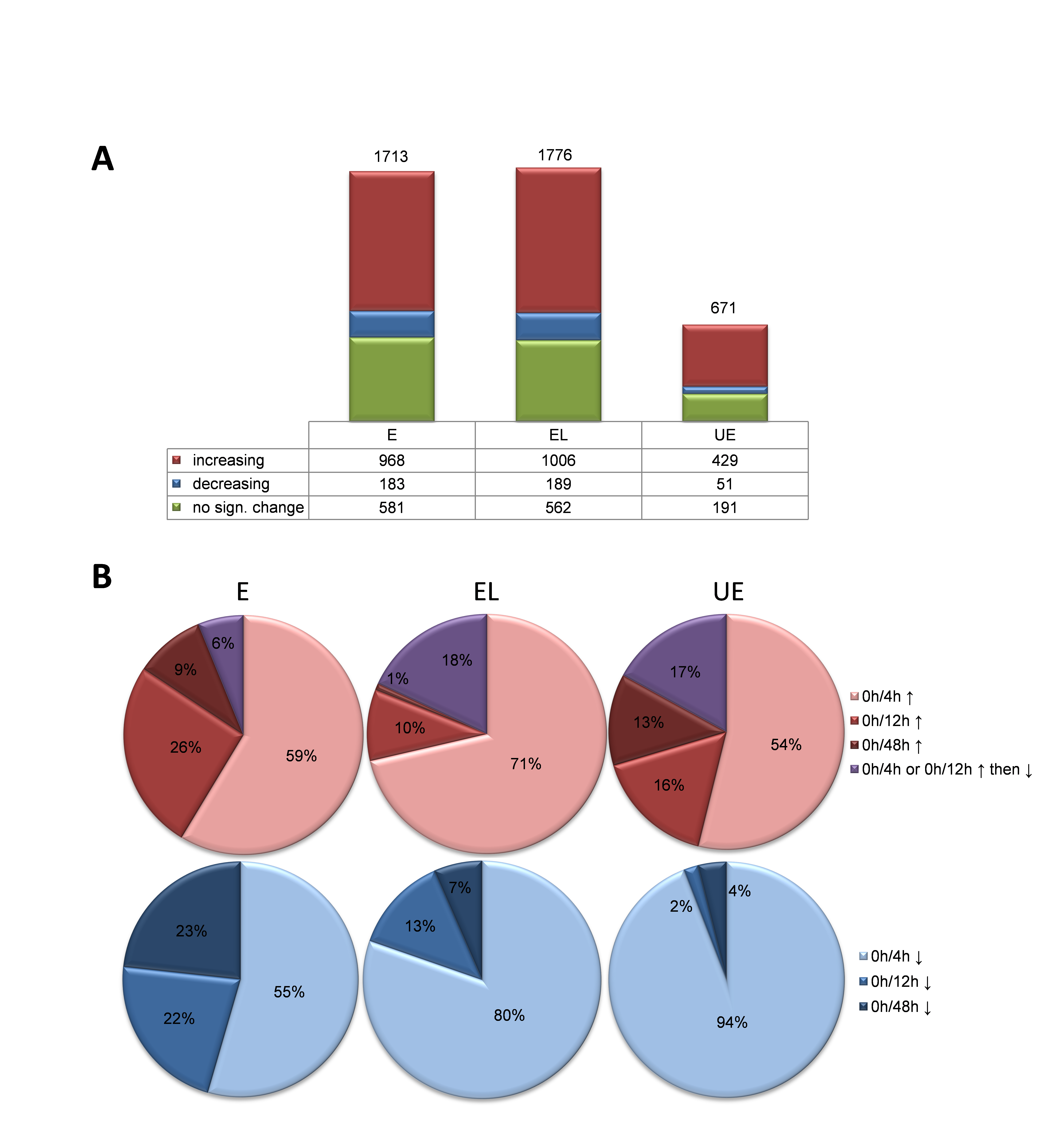

Supplement: Figure S5 — Number of metabolite markers, and the proportion of significantly changing markers found in each sample type. (A) The number of metabolite markers in each of the samples types after the data cleaning process, and number of significantly decreasing and increasing markers in each sample type. Marker was considered to be significantly changed if the value between two time points had fold change either <0.5 or >2, with p-value less than 0.05. (B) The distribution of the increasing (upper pie charts) and decreasing (lower pie charts) markers in the incubation after 4, 12, and 48 hours. Some of the markers were intermediate metabolites that were first increasing, but towards the end of the incubation were again decreasing, and these are shown on purple sectors in the upper pie charts. (TIF) [file pone.0039322.s005.tif]
