# Supplementary material for: In Vitro Microbiotic Fermentation Causes an Extensive Metabolite Turnover of Rye Bran Phytochemicals
Source: PLoS One. 2012 Jun 20;7(6):e39322. doi: 10.1371/journal.pone.0039322 (PMC3380017; doi:10.1371/journal.pone.0039322)
Supplement: Table S1 — MS/MS fragmentation of the key metabolites. Identification was done based on comparison with standards, or with earlier reported MS/MS results. (DOCX) [file pone.0039322.s006.docx]

**Supplementary Table 1**: MS/MS fragmentation of the key metabolites. Identification was done based on comparison with standards, or with earlier reported MS/MS results.

References

Hanhineva K, Rogachev I, Aura AM, Aharoni A, Poutanen K, Mykkanen H (2011) Qualitative characterization of benzoxazinoid derivatives in whole grain rye and wheat by LC-MS metabolite profiling. *J Agric Food Chem* **59:**921-927. doi: 10.1021/jf103612u

Hanhineva K, Rogachev I, Aura A, Aharoni A, Poutanen K, Mykkänen H Identification of novel lignans in the whole grain rye bran by non-targeted LC–MS metabolite profiling. *Metabolomics* 1-11. doi: 10.1007/s11306-011-0325-0

Redeuil K, Smarrito-Menozzi C, Guy P, Rezzi S, Dionisi F, Williamson G, Nagy K, Renouf M (2011) Identification of novel circulating coffee metabolites in human plasma by liquid chromatography–mass spectrometry. *Journal of Chromatography A* **1218:**4678-4688. doi: DOI: 10.1016/j.chroma.2011.05.050

Stoupi S, Williamson G, Drynan JW, Barron D, Clifford MN (2010) A comparison of the in vitro biotransformation of (?)-epicatechin and procyanidin B2 by human faecal microbiota. *Molecular Nutrition & Food Research* **54:**747-759. doi: 10.1002/mnfr.200900123
